# Supplementary material for: Effect of sampling frequency on fractal fluctuations during treadmill walking
Source: PLoS One. 2019 Nov 7;14(11):e0218908. doi: 10.1371/journal.pone.0218908 (PMC6837491; doi:10.1371/journal.pone.0218908)
Supplement: Attachment — Submitted filename: Response to reviewers.docx [file pone.0218908.s003.docx]

**Response to reviewers**

We thank the editor and reviewers for their comments. We have addressed each comment and revised the small typos.

**1) It seems from context that the statement on line 264-265 is specific to DS60, but this is not clear in the statement. Please clarify.**

We have clarified the statement as follow:

*This suggests that sampling motion capture at 60 Hz (i.e., as in condition DS60) may lead to less accurate α-DFA values, assuming condition 240 as the gold-standard.*

**2) Table 1: Leading zero missing for ICC 95% CI for Step Length [0.811-969] should probably be [0.811-0.969]**

Revised.

**3) Table 2: Leading zero missing for a-DFA Stride Time in Table 2 (.14) should be (0.14)**

Revised.
